# Impact of health systems factors on effectiveness of malaria case management in Sub-Saharan African countries

**DOI:** 10.1186/1475-2875-13-S1-P37

**Published:** 2014-09-22

**Authors:** Katya Galactionova, Fabrizio Tediosi, Don de Savigny, Thomas Smith

**Affiliations:** 1 Swiss Tropical and Public Health Institute, Basel, Switzerland; 2 University of Basel, Basel, Switzerland

## Background

Scale-up of malaria preventative and control interventions over the last decade resulted in substantial declines in mortality and morbidity of the disease. Sustaining these gains in the future will depend on the health system performance. Treatment provides individual benefits by curing infection and preventing progression to severe disease as well as community-level benefits by reducing the infectious reservoir and averting the emergence and spread of drug resistance. However many patients with malaria do not access care, providers do not always comply with treatment guidelines, so patients do not necessarily receive the correct regimen. Even when the correct regimen is administered there is concern that patients will not adhere, leading both to treatment failures and potentially to the spread of drug resistance.

## Methods and results

We propose a framework to assess the effective-ness of malaria case management and apply it to derive estimates for 42 high-burden Sub-Saharan African countries. We explicitly consider implications of treatment seeking, source of treatment, systems compliance with the recommended antimalarial treatment, adherence with the drug regimen, and quality of the antimalarial medication on effectiveness of malaria case management. Country-specific parameters are populated from the Demographic and Health Surveys and other published sources. We assess the relative importance of these factors on the level of effective coverage within a given setting and the variation in these systems indicators across the region. Our findings suggest that overall only about two-thirds of fevers for which treatment is sought are treated effectively; that is about 40% of treatments fail to clear parasitaemia. There is however a lot of variation in the effectiveness of malaria case management across the region. Different factors account for inefficiencies in different countries.

## Conclusions

The patterns of inter-country variation suggest that these system failures are amenable to change. Identifying and remedying these should become key priority areas for malaria control policy in the region. Significant losses in efficacy of treatment are found even in countries with a relatively high quality of care. In general there is a need for more valid, reliable, and representative data on these important quantities. Treatment is an important determinant of the burden of malaria disease, and of the incremental benefits of other interventions, but has often been disregarded in resource allocation decisions. Including these aspects would allow policy makers to make decisions on the basis of the specific conditions of the settings where malaria control interventions are actually needed.

